# British Hospitals Association Conference

**Published:** 1924-07

**Authors:** 


### BRITISH HOSPITALS ASSOCIATION CONFERENCE.

### AN IMPRESSION.

### BY A RANK-AND-FILE MEMBER.

Victory House, Leicester Square, the meeting-place of the British Hospitals Association's Conference' was at one time the Victory Hotel, and before that the Café de l'Europe of our youth. But no more harmonious gathering was ever held within its walls, now devoted to less material things, than during the sessions of the Conference. On the morning of the first day, Thursday, June 19th, the large hall on the first floor was almost full, the attendance being quite up to the Sheffield standard of last year. The only spot of colour within the room was the handsome robe of office of Sir Humphry Rolleston, K.C.B., the President of the Royal College of Physicians, who in a graceful manner welcomed the delegates. The President of the Association, Sir Arthur Stanley, was in the Chair, and indeed no gathering of this kind would be complete without his genial countenance.

#### A Startling Announcement.

The first paper was read by Dr. F. N. Kay Menzies, the new director of Hospital and Medical Services for the British Red Cross Society. We have rarely listened to a paper so full of material and so bare of useless rhetoric. Within a very small space it was a complete *résumé* of the health services of the country, and, as afterwards appeared, was a sufficient inspiration to the meeting to call upon the Association to endeavour to produce themselves, or to induce some other body to issue a full and accurate survey of all essential details of State, Municipal and Voluntary services in this country for the prevention and cure of disease. Dr. Menzies made the startling announcement, well supported by the facts in his paper, that in his opinion at least four-fifths of the treatment available for the sick in the Metropolitan area is provided for out of rates and taxes. A useful discussion followed his paper, in which Viscount Hambleden, Lord Armstrong, Colonel Macintosh and others took part. In the mid-day recess the company was genially entertained at luncheon by the Proprietors and Editor of the Hospital and Health Review. Mr. J. Penderel-Brodhurst was, of course, in the Chair, and very wisely kept the few speeches that followed the luncheon to narrow limits.

#### Criticising the Nursing Council.

In the morning a loyal message had been sent by the members present to the King, and at the commencement of the afternoon session a gracious reply was read. Then Sir Wilmot Herringham gave an extempore address on the training of nurses. Apart from his long association with St. Bartholomew's Hospital, Sir Wilmot's position as Chairman of the General Nursing Council added weight to his remarks. He briefly described the history of nursing since the Sarah Gamp days, and then dwelt with the efforts of the General Nursing Council to keep up the prestige of the nursing profession, which, he thought, could by no other means attract women as it did when so few professions were open to them. It was a grand opportunity for critics of the Nursing Council, and there were present some delegates, chiefly representatives of small hospitals, which, by reason of one feature or another, did not, acting without the co-operation of another, hospital, fulfil the requirements of the Nursing Council as training schools. These gentlemen expressed their views. Mr. Eason, the Medical Superintendent of Guy's Hospital, considered that the movement for State Registration and the labours of the General Nursing Council were the biggest things in the way of bringing hospitals together which had been done during the last twenty or thirty years. He described how Guy's was being affiliated with several small hospitals of a special character.

#### Hours of Ease.

Viscountess Hambleden gracefully welcomed the members of the Conference at 3, Grosvenor Place, and in a surprisingly short time the delegates had all changed their clothes, donned their orders and decorations, and were seated in the Great Gallery of the Royal Automobile Club to partake of the excellent dinner that had been provided. It may here be said, as was so often said during the course of the evening, that the organisation of the Conference had been extremely well carried out, the main part of the work naturally falling upon the Secretary of the Association, Mr. J. Courtney Buchanan, C.B.E. The speeches after the dinner were of a high order. and Lord Knutsford quite adequately supplied the part which is usually taken by professional entertainers in proposing the health of the Chairman. He said that when the matter of the speeches had been previously discussed he had offered to sing, but the Executive Council immediately decided that there should be no music during the dinner. We have never heard Lord Knutsford sing, but if he can do so as entertainingly as he can speak he must be an artist in this as well as in the task of raising money. The popular Head of the London Hospital said that he had always advised younger chairmen that a good chairman must be a gentleman with the instincts of a felon, and this description he thought might well apply to Lord Hambleden.

#### Approved Societies and Voluntary Hospitals.

The second day of the Conference began with the annual business meeting, which passed off without any notable incident. Mr. R. J. Meller, M.P., of the Prudential Insurance Society, then read a paper on "Approved Societies in Relation to Voluntary Hospitals." The information he gave the meeting contained much interesting detail, and it was foreshadowed that the surplus at the next quinquennial valuation of Approved Societies would perhaps be even larger than that at the last.

#### Strenuous Days.

The members of the Council and a number of other visitors were entertained to luncheon by several of the City Companies, and in the afternoon, by arrangement, visitors from the Provinces were received at a number of general and special hospitals.

[The papers read at the Conference are printed on the ensuing pages.]

